# Diagnostic accuracy of diffusion weighted MRI in differentiating benign and malignant meningiomas

**DOI:** 10.12669/pjms.35.3.1011

**Published:** 2019

**Authors:** Din Muhammad Sohu, Saba Sohail, Rehana Shaikh

**Affiliations:** 1 *Dr. Din Muhammad Sohu, MBBS, FCPS-II Resident, Department of Radiology, Dow University of Health Sciences/ Dr. Ruth K. M. Pfau, Civil Hospital, Karachi, Pakistan*; 2 *Dr. Saba Sohail, MBBS, MCPS, FCPS, Ph.D, Department of Radiology, Dow University of Health Sciences/ Dr. Ruth K. M. Pfau, Civil Hospital, Karachi, Pakistan*; 3 *Dr. Rehana Shaikh, MBBS, FCPS, EDIR, Department of Radiology, Dow University of Health Sciences/ Dr. Ruth K. M. Pfau, Civil Hospital, Karachi, Pakistan*

**Keywords:** Benign meningiomas, Diagnostic accuracy, DWI, Histopathology, Malignant meningiomas

## Abstract

**Objective:**

To determine the diagnostic accuracy of DWI in differentiating benign and malignant meningiomas keeping histopathology as gold standard.

**Methods:**

This was a descriptive analytical study conducted at Radiology Department, DUHS/Dr. Ruth K. M. Pfau Civil Hospital Karachi, from August 2016 to March 2018.It included152 patients clinically suspected of meningioma on conventional neuroimaging. Imaging features of DWI were compared with histopathology findings. The diagnostic accuracy of DWI was calculated in terms of sensitivity, specificity, accuracy, PPV and NPV using histopathology as gold standard.

**Results:**

There were 59 male and 93 female patients with mean age of 55.38±9.8 years. Mean duration of sign and symptoms was 5.67±2.57 months. Out of 152 patients, 117(77%) and 35(23%) were differentiated into benign and malignant meningiomas respectively by DWI while 135(88.82%) and17(11.18%) patients were diagnosed respectively on histopathology. The sensitivity, specificity, PPV, NPV and accuracy of DWI of 84.4%, 82.3%, 97.4%, 40%, and 84.2% respectively keeping histopathology as gold standard.

**Conclusion:**

DWI features along with calculation of ADC values is a reliable non-invasive technique for differentiating benign and malignant meningiomas. However the low negative predictive value necessitates the use of histopathology.

## INTRODUCTION

Meningiomas are the most common primary extra-axial non-glial intracranial tumors, comprise approximately 14–20% of all intracranial tumors.^1^,^2^ Meningiomas commonly occur on the brain surface and rarely in the brain ventricles. Mostly they are seen in middle aged patients showing female predilection with male:female ratio of 1:2.^3^ Tumors less than 2.5cm are rarely symptomatic whereas, larger tumors show symptoms which worsen with time.^4^

Most of meningiomas are typically benign, slow growing and curable by surgery depending on location.^5^ About 10% of meningiomas are atypical or malignant associated with higher morbidity and mortality. They may invade the adjacent bone and brain parenchyma so prone to recur in 29% - 41% of patients.^6^ So it is important to distinguish them correctly for treatment planning, deciding the aggressiveness of surgical resection and the need of combined radiation therapy.^2^,^7^ Though some radiological features on conventional neuroimaging like intratumoral cystic change, hyperostosis of the adjacent skull, bony destruction, extracranial tumor extension through the skull base, arterial encasement, and peritumoral brain edema have been found to distinguish these two entities; no single feature has been found to be highly reliable.^8^

Diffusion weighted imaging (DWI) is a non-invasive technique, based on the measurement of water diffusion in tissues, which provides information about tissue microstructures, important in the grading of tumors before surgery.^9^ Few previous studies have found that the atypical/ malignant meningiomas tend to be markedly hyperintense on diffusion weighted images (DWI) and exhibit markedly decreased value on apparent diffusion coefficient (ADC) imaging when compared with normal brain parenchyma, while the benign meningiomas have a variable appearance on diffusion weighted images and tend to have higher ADC values compared with normal brain.^6^,^9^,^10^ However these already conflicting results needs to be validated in our population, where tuberculomas are common confounders for the meningiomas and may give a similar appearance with caseous material simulating signals of calcification on screening MRI. So this study was conducted to compare diffusion-weighted imaging findings of different meningiomas by using apparent diffusion coefficient (ADC) values for predicting tumor grade into benign and malignant meningiomas.

## METHODS

It was a descriptive analytical study conducted at CT & MRI Centre, Dow University of Health Sciences/Dr. Ruth K. M. Pfau Civil Hospital Karachi, from August 2016 to March 2018. Inclusion criteria were patients of either gender between 20-70 years of age, primarily suspected of meningioma on clinical features and conventional cross sectional imaging either on MRI or CT scan in last 12 weeks, and underwent DWI at the study centre. Patients who had claustrophobia, history of indwelling metallic implants and cardiac pacemakers, post-operative or recurrent meningiomas were excluded from the study.

Sample size was calculated by taking expected sensitivity 72.9% and specificity 73.1% of DWI^9^ with desired precision of 0.10, 35% prevalence^11^ and 95% confidence level. The total calculated sample size was 152. Written informed consent was obtained from each subject and permission was also obtained from The Institutional Review Board.

Diffusion Weighted images (DWI) were obtained using a single-shot echo planar spin echo technique (TR/TE/NEX: 4200/140 ms/I) with diffusion sensitivities of b values = 0, 500 and 1000 s/mm^2^ on a 1.5-Tesla MR scanner (GE Health Care Signa H D). The diffusion gradients were applied sequentially in three orthogonal directions (X, Y and Z directions). The scanning parameters were 5 mm slice thickness, 1mm interslice gap, 240mm FOV and a matrix of 128 x 256 with 80s total acquisition time. Three types of images were obtained; orthogonal images, trace images and ADC maps. The ADC maps were calculated automatically by MRI software and included in the sequence. ADC values were measured in 10^− 3^mm^2^/s by keeping different regions of interest (ROI) in the lesion and contralateral region.

Images were analyzed and reported as benign or malignant meningiomas according to DWI using ADC values and then compared with histopathological diagnosis obtained later after tumor resection at the Neurosurgery department of the same hospital.

Data collected was analyzed by SSPS program version 20. Mean and standard deviation were calculated for quantitative variables like age and duration since diagnosis on conventional MRI/CT scan. Frequency and percentages were calculated for qualitative variables like gender, diagnosis on DWI and histopathological diagnosis were calculated. The diagnostic accuracy of Diffusion weighted MRI for differentiating benign and malignant Meningiomas was calculated in terms of sensitivity, specificity, positive predictive values and negative predictive values keeping histopathology as gold standard. Post-stratification 2x2 table was generated to calculate these parameters.

## RESULTS

One hundred and fifty two patients were enrolled to determine the diagnostic accuracy of Diffusion Weighted MRI in differentiating benign and malignant meningiomas keeping histopathology as gold standard. There were 59 males and 93 females, aged from 21 to 70 years with mean age of 55.38 ± 9.8 years. The mean duration of sign and symptoms of study subjects was 5.67 ± 2.57 months while the mean duration after primary diagnosis of meningioma on conventional CT / MRI till differentiation on diffusion weighted MRI of study subjects was 4.7 ± 2.5 weeks.

**Fig.1:**
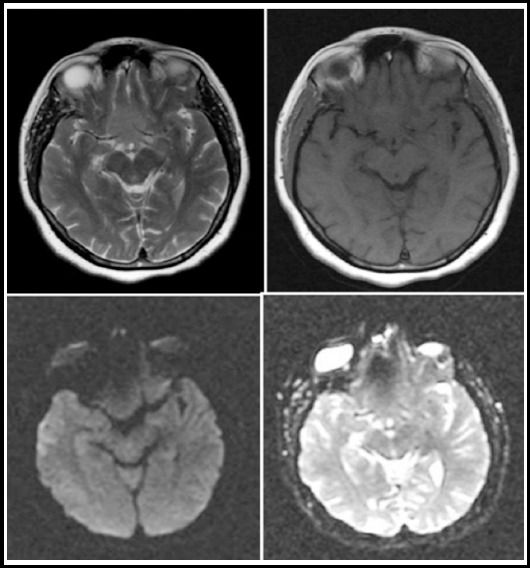
Axial T2WI, T1WI, DWI and ADC images show a benign suprasellar meningioma without diffusion restriction on DWI/ADC.

**Fig.2:**
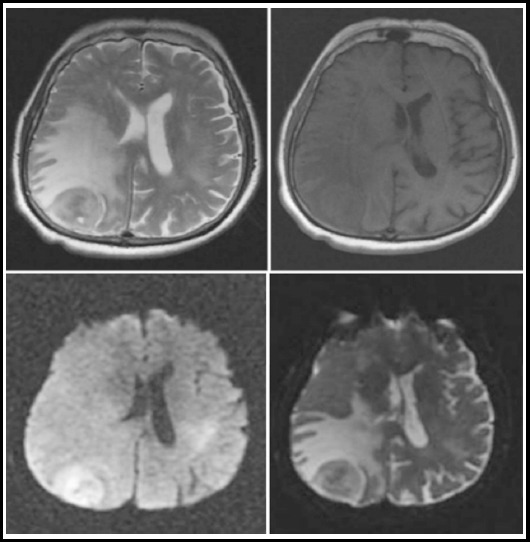
Axial T2WI, T1WI, DWI and ADC images reveal a malignant posterior parietal meningioma showing diffusion restriction on DWI/ADC and marked peritumoral edema.

Out of 152 patients, 117 patients (77%) showed benign meningiomas; while 35 patients (23%) showed malignant meningiomas on diffusion weighted MRI (DWI) using ADC values (Fig. 1 and 2). While 135 (88.82%) were found to be benign meningiomas and 17 (11.18%) as malignant meningiomas on histopathology. Twelve patients out of 17 having malignant meningioma were seen in male patients.

So 114 patients were correctly differentiated by DWI using ADC values as benign meningioma and 14 patients as malignant/atypical meningiomas when compared with histopathology resulting in sensitivity of 84.4%, specificity of 82.3%, PPV of 97.4%, NPV of 40% and accuracy of 84.2% (Table-I).

**Table-I:** Diagnostic accuracy of DWI with Histopathology as Gold Standard to differentiate benign and Malignant Meningiomas (n= 152).

Benign (n = 135)		Histopathology			P-value

		Malignant (n = 17)	Total		
DWI/ADC	Benign (n = 117)	114	3	117	P<0.001
	Malignant (n = 35)	21	14	35	
	TOTAL	135	17	152	
	Sensitivity	Specificity	PPV	NPV	Accuracy
	84.4%	82.3%	97.4%	40%	84.2%

Chi square test was applied, P-Value ≤0.001 considered as significant.

## DISCUSSION

Meningiomas are common but often an incidental finding on neuroimaging. The benign meningiomas are promptly diagnosed but their differentiation from atypical/malignant tumors by using conventional MRI is still quite difficult. Neuroimaging features like heterogeneous signals and enhancement, perilesional edema, and irregular cerebral surface are not unique and reliable to diagnose malignant meningiomason conventional MRI. For the surgical and treatment planning a diagnostic method is highly desirable for accurate distinction between benign and malignant meningiomas.^12^

Diffusion weighted imaging (DWI) along with the calculation of apparent diffusion coefficient (ADC), is a reliable and non-invasive technique of choice for accurate assessment and in treatment planning of different types of brain tumors. It has more advantages in the distinction and differentiation of benign from malignant meningiomas on the basis of ADC values.^12^

Several studies are available that characterize meningioma by DWI, however the provided data was inconsistent.^6^,^10^,^13^-^15^ Some did not identify any significant difference between the mean ADC ratios of benign and atypical/malignant tumors^13^ while some studies found that the mean ADC value of benign tumors was significant higher than the ADC value of atypical/malignant meningiomas.^6^,^10^,^14^,^15^ So this study was done to assess the overall performance of the DWI in terms of sensitivity, specificity, positive predictive value (PPV), negative predictive value (NPV) and accuracy in our population.

In this study, the mean age of patients was 55.38±9.8 years which is comparable to Kane AJ et al.^16^ and Ignjatovic J et al.^17^ that showed mean age of patients was 54 years and 53 years. In the current study, meningiomas particularly benign were found more frequent in females but malignant meningioma were more commonly observed in male, which is corroborating the reports from Samadi N et al.^18^ and Kane AJ et al.^16^

The benign meningioma showed variable appearance like hypointense, isointense and slightly hyperintense on DWI and ADC maps with ADC values of more than 0.85×10^-3^mm^2^/sec while malignant/atypical meningiomas returned hyperintense signals on DWI and hypointense in ADC maps, with ADC values less than 0.85×10^-3^mm^2^/sec. Similar signals were demonstrated by Khedr SA et al.^2^ and Liu Y et al.^19^ in their studies. Liu Y et al.^19^ also found that hyperintensity of lesion on DWI as the strongest independent predictor of high grade meningioma.

This study showed 84.4% sensitivity of DWI which was comparable with the study done by Tantawy HI et al.^20^ (83.3%) but was higher than Suruv A et al.^15^ (72.9%) and less than Nagar VA et al.^14^ (96%). The specificity of DWI was 82.3% in this study which was comparable to the studies by Nagar VA et al.^14^ (82.6%) and Bano S et al.^12^ (83.2%) but higher than the study by Todua F et al.^21^ (80.0%) and Suruv A et al.^15^ (73.1%) in differentiating meningiomas. Our study showed higher positive predictive value than by Tantawy HI et al.^20^ (83.3%) and Nagar VA et al.^14^ (85.7%).

Our study also showed overall better results except the negative predictive value than a study done by Surov A et al.^9^ who determined the sensitivity of 72.9%; specificity of 73.1%; accuracy of 73.0%; positive predictive value of 33.3% and negative predictive value of 96.8%, respectively taking ADC_mean_ value of less than 0.85 × 10^− 3^ mm^2^s^− 1^ to differentiate between benign and atypical/malignant meningiomas.

Most previous studies have showed variable negative predictive value ranging from 68.3% to 96.8% to distinguish benign and malignant meningiomas by DWI.^9^,^12^,^15^,^20^ While our study showed low negative predictive value of 40%, which may be either due to different study population, variation in age and gender of study population or small sample size of study population. Another reason may include the necrosis of malignancy that may be mistaken for cystic change of benign etiology on DWI alone. So despite good accuracy, sensitivity and specificity DWI MRI negative for malignancy still needs to be confirmed with histopathology.

### Limitations in this study

It was small sample size and the study was confined to single centre. Another limitation was the low negative predictive value (NPV) in this study likely to be due to varying tumor morphology, which warrants further research on larger population.

## CONCLUSION

A DWI MRI scan has a high sensitivity, specificity and accuracy; but a negative scan suggesting benign disease has to be interpreted with caution due to low negative predictive value. Histopathology should not be omitted in such cases.

### Author`s Contribution

**DMS** conceived, designed and did data collection, statistical analysis & manuscript writing.

**RS** designed and did editing of manuscript.

**SS** did review and final approval of manuscript.
